# Validation of Capturing Sleep Diary Data via a Wrist-Worn Device

**DOI:** 10.1155/2015/758937

**Published:** 2015-12-15

**Authors:** Carla R. Jungquist, John J. Pender, Karen J. Klingman, Jamie Mund

**Affiliations:** ^1^University at Buffalo School of Nursing, University at Buffalo, South Campus, 202 Beck Hall, 3435 Main Street, Buffalo, NY 14214, USA; ^2^SUNY Upstate Medical University, Syracuse, NY 13210, USA

## Abstract

Paper sleep diaries are the gold standard for assessment of sleep continuity variables in clinical practice as well as research. Unfortunately, paper diaries can be filled out weekly instead of daily, lost, illegible or destroyed; and are considered out of date according to the newer technology savvy generations. In this study, we assessed the reliability and validity of using a wrist-worn electronic sleep diary.* Design.* A prospective design was used to compare capturing 14 days of sleep continuity data via paper to a wrist-worn electronic device that also captured actigraphy data.* Results.* Thirty-five healthy community dwelling adults with mean (sd) age of 36 (15), 80% Caucasians, and 74% females were enrolled. All sleep continuity variables via electronic and paper diary capture methods were significantly correlated with moderate, positive relationships. Assessment of validity revealed that electronic data capture had a significant relationship with objective measure of sleep continuity variables as measured by actigraphy. Paper diary variables were not significantly associated with objective measures.* Conclusions*. The use of a wrist-worn device to capture daily sleep diary data is as accurate as and for some variables more accurate than using paper diaries.

## 1. Background

Capturing subjective sleep continuity data from patients in clinical practice and participants in research is often recorded on paper diaries [1]. Experience has shown that getting a patient or participant to fill out the diary in the evening and upon awakening for the day proves challenging. Often paper diaries will (1) be soiled or illegible, (2) have data reported in ranges as opposed to specific data points, or (3) be completely filled out on the day of the office visit [2]. Without accurate data, it is difficult for the therapist to assess the patient's sleep and prescribe effective treatment. In recent years, web based programs and applications on hand-held devices have shown promise in improving accuracy of data collection. Actigraphy has also become a popular and valid and reliable [3–5] avenue of collecting objective measures of sleep. With the evolution of electronic devices and the proclivity for their use, it seems only natural for scientists and clinicians to gravitate in this direction. Now that the technology is available for use it is important for scientists to validate their measurement capabilities.

In this study, we assessed the reliability and validity of using a wrist-worn electronic device to capture subjective sleep diary data. We hypothesized that data collected via electronic and paper capture would differ, in that electronic capture would have a stronger correlation to objective (actigraphy) data. We also hypothesized that the data captured electronically would be more legible and convenient for the participants.

## 2. Methods

A within-participants descriptive design of 35 community dwelling healthy adults was used to compare capturing sleep continuity data via paper to a wrist-worn electronic device that also captured actigraphy data. This study was nested within a larger study to validate the sleep screening questions on the Behavioral Risk Factor Surveillance System (BRFSS) by the Centers for Disease Control and Prevention. The study was reviewed and approved by the University at Buffalo Social and Behavioral Sciences Institutional Review Board.

*Study Procedures*. Thirty-five consecutively enrolled participants were asked to fill out paper diaries each morning on awakening for 14 consecutive days. They also wore the PRO-diary device on their nondominant wrist for the same 14 consecutive days. The study consisted of one study visit at the beginning of the 14 consecutive days. During the study visit, after reviewing and signing informed consent, participants responded to questionnaires used to characterize the sample. During the 14-day period, participants wore the Apnealink device for one night to screen for obstructive sleep apnea.

Exclusion criteria included (1) being under the age of 18 and (2) wearing positive airway pressure or oxygen during sleep. Recruitment strategies included posters, Craig's list, Research Match, and word of mouth.

### 2.1. Measures

*Participant Characteristics*. Sex, age, race, ethnicity, years of education, employment status, annual individual income, medical conditions, medications, and shiftwork status were the self-reported data collected on each participant.

*Sleep, Mood, Pain, and Physical and Social Function Related Variables Questionnaires*

*Insomnia Severity Index (ISI)*. The ISI is a commonly used standard instrument that assesses sleep disturbance and quality of sleep. The total score of the 7 questions was used in the analysis. The score ranges from 0 to 28, and above 7 is considered clinically relevant insomnia [6, 7].

*PROMIS-57 Profile*. This profile questionnaire consists of 57 valid and reliable [8–11] questions assessing 7 components: physical function, social function, pain, sleep, anxiety, depression, and fatigue. Timeframe referenced by the questions was the past 7 days. Each component consists of 8 questions scored 1–5 with total component scores ranging from 8 to 40. The higher the score, the more the symptoms or the higher the functioning. There is one question on pain severity that is scored 0–10. When *t*-scores are calculated on each component, the average score for the population is 50.

*Epworth Sleepiness Scale (ESS)*. This questionnaire measures the degree to which the person is likely to fall asleep in 8 situations. The total score ranges from 0 to 24 and a score of >10 is considered clinically relevant [12].

*Device*. The Patient Reported Outcome-diary (PRO-diary) device manufactured and marketed by Camntech was used in this study [13]. All devices were purchased and the manufacturer was not aware of nor had any influence on this study. Participants were asked to wear the PRO-diary that includes actigraphy for 14 consecutive days. The diary software and the actigraphy software do not communicate with each other and were downloaded separately into an Excel database. Each diary entry is time-stamped for the time data was entered. Questions for the diary (same as paper diary questions) were developed within a database and uploaded to the device to allow for unique questionnaire structure. Our sleep and daytime function questionnaire was adapted from the consensus sleep diary [14]. Participants were instructed to wear the device on the nondominant wrist at all times except when in the shower. Before going to bed and again on awaking in the morning, they initiated the electronic diary by tapping lightly on the button. The questions would then appear one at a time. The participant was instructed to enter their responses using the touch slider. The motionware portion of the device collected data continuously in one-minute epochs.

*Paper Sleep Diaries*. Participants were instructed in a standardized manner on filling out sleep diaries upon waking in the morning for a period of 14 consecutive days. Questions inquired about (1) time to bed, (2) time out of bed, (3) number of times awake during the night, (4) minutes it took to fall asleep, (5) minutes awake during the night, and (6) amount of time out of bed during the night. Variables derived from the paper diaries were calculated two-week averages for all sleep continuity variables (minutes to go to sleep, minutes awake during the night, and number of awakenings).

### 2.2. Data Management and Analysis

Questionnaire and sleep diary data that were captured via paper were transcribed and entered into an Excel spreadsheet by a research assistant and then imported into SPSS version 21 for analysis. A second research assistant verified transcribed data. Illegible data entries on the paper documents were coded as missing data. Data from the devices were downloaded into an Excel spreadsheet, cleaned, formatted, and imported into SPSS for analysis. Variables representing sleep efficiency and total sleep time were calculated on the paper and electronic captured data. The Motionware software for actigraphy data calculates SE and TST automatically. Variables were assessed for normality and found to meet the assumptions of Pearson *r* correlation analysis and *t*-tests. Analysis strategies included descriptive statistics as well as Pearson *r* correlations and paired *t*-tests between scores on sleep latency (SL) in minutes, wake after sleep onset (WASO) in minutes, and number of awakenings (NWAK) via the paper diary, electronic diary, and actigraphic data. Bland-Altman procedures were used to assess differences between paper and electronic diary capture on SL, WASO, and NWAK. Results were then compared to objective data to determine whether paper or electronic capture was closer to objectives measures.

*Missing Data*. Ten participants did not complete all 14 days of data via the electronic or paper diary. To account for differences in number of days of data, data was averaged over the total number of actual data points. Specifically, participants who filled out only 3 of the 14 days of data were included in the analysis and average of those 3 days was used in the analysis.

## 3. Results

*Participant Characteristics*. Thirty-five participants completed all study procedures (Table 1). The majority of the participants were females with 32 percent reporting an income of less than $30,000 a year and 47 percent reporting an income between $50,000 and 100,000 a year. Education level ranged from high school to graduate education. Overall physical and social functioning were high with little depression, anxiety, pain, excessive daytime sleepiness, or fatigue. The apnea hypopnea index ranged from 0 to 12 events per hour. There were seven subjects with mild sleep apnea (AHI ≥ 5) as diagnosed by a sleep specialist. Fifty-two percent of the participants reported at least mild insomnia (Insomnia Severity Index total score > 7).

*Description of Data Recorded Comparing Paper versus Electronic Capture*. In this study there were a total 490 possible days of data entry (calculated by multiplying 35 subjects by 14 days). There were 25 subjects that completed 14 days of data collection, 2 completed 10 days, 1 completed 11 days, 2 completed 12 days, and 5 completed 13 days for a total of actual data entry on 470 days over the course of this study. Of the possible 470 days to complete their data entry, we received data via paper dairy on 470 days and electronic entry on 420 days. There was one outlier in this study that only wore and interacted with the PRO-diary device on 3 of the 14 days requested. This participant also filled out their paper diary with numbers on the first day and then drew a line through to the last day to present carrying forward the exact same numbers for each night. Four paper dairies were returned with stains but remained legible. Two participants wrote ranges as their estimation of number of awakenings and minutes awake. In visually reviewing the times when the morning electronic diary data was entered, it was obvious that several entries were made as many as 12 hours after actual waking time.

*Differences between Paper and Electronic Diary Data*. See Table 2 for descriptive results. In general, minutes to fall to sleep (SL) and minutes awake after sleep onset (WASO) were reported to be higher via electronic diary capture when compared to paper. To assess for the agreement between paper and electronic capture of subjective diary data, *t*-test analysis was performed on SL, WASO, and NWAK. Significant differences were found on SL [mean difference (sd): 4.90 (13) minutes, *t* = 2.20, df 34, *p* = .04 (95% CI 0.36–9.45)] and WASO [mean difference: 10.91 (10), *t* = 6.34, df 34, *p* < .01 (95% CI 7.42–14.41)]. No significant difference was found in number of awakenings during sleep (NWAK). Bland-Altman comparisons were also performed on above variables.

**Figure 1:**
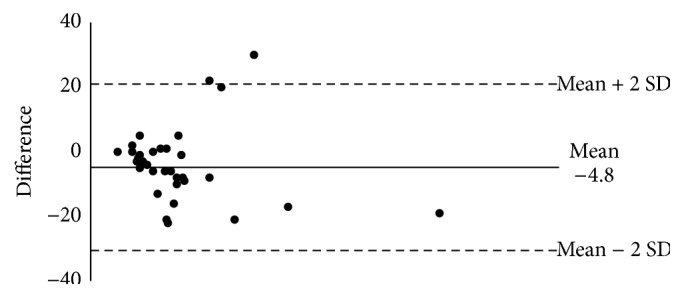
Bland-Altman of PRO versus Paper sleep latency 2-week avg. (*n* = 35).

The Bland-Altman comparison of paper and electronic capture of sleep latency data indicates that the lack of agreement is −4.8 with SD 11 (95% CI −25–18) (Figure 1). The lack of agreement is around 3.7 minutes between paper and electronic capture but could differ as much as 25 minutes.

**Figure 2:**
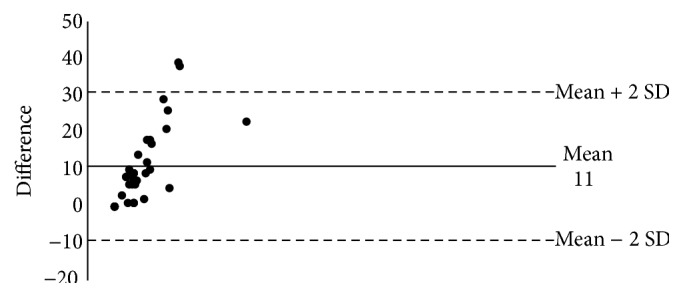
Bland-Altman PRO versus paper WASO (*n* = 35).

The Bland-Altman comparison of paper and electronic capture of minutes awake after sleep onset (WASO) data indicates that the lack of agreement is 11 with SD 10 (95% CI −9.2–31) (Figure 2). The lack of agreement is around 11 minutes between paper and electronic capture but could differ as much as 31 minutes.

**Figure 3:**
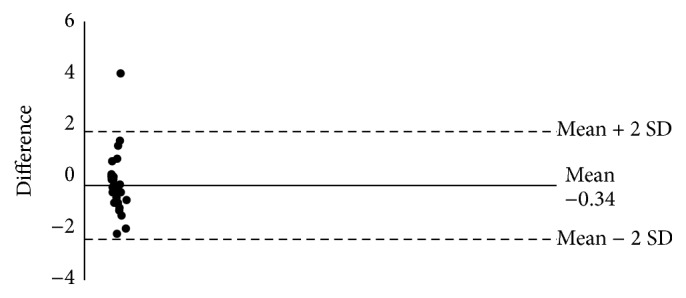
Bland-Altman of PRO versus Paper NWAK 2-week avg.

The Bland-Altman comparison of paper and electronic capture on number of awakenings (NWAK) data indicates that the lack of agreement is −0.34 with SD 1.1 (95% CI −2.4–1.7) (Figure 3). The lack of agreement is around .34 awakenings between paper and electronic capture but could differ as much as 2.4 times.

*Differences between Electronic Diary Data and Actigraphy Data*. To assess if electronically captured subjective data was similar to objective sleep data, correlations were performed with actigraphy data. Correlations were significant and relationships were small-to-moderate on sleep latency (SL) and waking after sleep onset (WASO). See Table 3.

*Differences between Paper Diary Data and Actigraphy Data*. To assess if paper captured subjective data was similar to objective sleep data, correlations were performed with actigraphy data. See Table 4. There were significant small-to-moderate positive relationships on variables of SL and WASO. There was a significant moderate positive relationship on the calculated variable of total sleep time.

## 4. Discussion

In this study of diverse community dwelling healthy residents, sleep continuity data captured via paper versus electronic device was found to minimally differ, but electronically captured data was more closely aligned with objective data captured via actigraphy (Motionware software).

The use of subjective report of how a person has been sleeping is standard of care and considered an accurate and feasible method to screen for sleep problems as well as to diagnose insomnia and measure patient improvements when undergoing treatment [15]. With the advent of actigraphy, the need for subjective data has been questioned. Actigraphy data is derived from physical movement and software is used to estimate sleep parameters from the body movement. As evident in this study as well as other studies, actigraphy often overestimates awakenings and time awake during the night as compared to subjective report. Additionally, in patients with restless legs syndrome and obstructive sleep apnea, actigraphy data is highly likely to overestimate sleep disturbance [5, 16–18]. Additionally, the patient's perception of their sleep and how it is affecting their daytime function often triggers the patient to seek help from their primary care provider [19]. Therefore, it is always important to ask the patient or participant about their perception of their sleep and the use of sleep diaries is often wanted especially in a sleep practice.

When collection of sleep continuity variables is warranted, it is imperative that the method be convenient, feasible, efficient, reliable, and valid. One of the challenges with using paper diaries is the lack of certainty of when the data was recorded. It is well known that subjective estimates of minutes to get to sleep, minutes awake during the night, and the number of awakenings are more accurate if recorded immediately upon waking for the day [1]. The PRO-diary device data entries are time-stamped. In this study it was easy to look at when the participants entered their morning data. Entries often occurred later in the day or around usual bedtime. This seems reasonable as sometimes people may forget to enter data exactly when arising from bed but may remember later in the day. The PRO-diary device only allows for entries on the same day. When the calendar day changes, the participants will not be able to enter the previous days data. Therefore, data captured will be more accurate as recall of a night's sleep wanes after 24 hours. Whereas, on the paper diary, participants are able to enter data at any time point and often wait until directly before their appointment with their provider to fill out the paper diaries.

Providing a wrist-worn device to electronically capture data has been shown in this study to improve the likelihood of data that is more comparable to objective sleep data. The wrist-worn device that is always on the wrist is considered more convenient as the patient does not need to find the paper diary and pencil. Additionally, having objective (actigraphy) data to compare to subjective data can be helpful and (1) lead to the determination of and need for screening for periodic legs movements of sleep or sleep apnea, (2) assess for sleep state misperception, and (3) provide objective feedback for the patient during treatment. Another advantage of the device used in this study was the availability of an alarm that can be set to remind the patient to record their data. In this study, we did not set the device to alarm as, when set, the device will sound at the same time every day. In the future, perhaps the device companies may decide to program an alarm that will sound once the patient has been active for 10–15 minutes after a long period of sleep.

Another challenge when using paper diaries is legibility. In this study 2 of the 35 participants presented paper sleep diaries with illegible or missing data. Other participants recorded their data in ranges as opposed to a set number of minutes or awakenings. Ranges as opposed to a set data point are difficult to interpret when calculating instructions for sleep therapy or when making a diagnosis of insomnia. Most clinicians consider more than 30 minutes of SL and/or WASO with daytime complaints the criteria for diagnosis of insomnia. Participants' reporting ranges leads to imprecise parameters to base diagnosis and therapy. The wrist-worn device allowed for specific responses in 1-minute increments.

In this study, we found that paper and electronically captured data were significantly different on variables of SL and WASO. These mean differences were not clinically meaningful. When prescribing behavioral sleep therapy, the clinician will often prescribe bedtimes and waking times in 15- or 30-minute increments [20]. When diagnosing insomnia, 30 minutes in bed awake during the night is the clinical cutoff most used. But if a patient has significant daytime complaints and is bothered enough by being awake 25 minutes while intending to be asleep, it is likely that the diagnosis will be made and therapy will be prescribed. Therefore, a difference between electronic and paper capture of diary data of 4 minutes on SL or 12 minutes on WASO may not be clinically relevant.

In this study, we also found that electronically captured data may be more closely aligned with objective data on variables of SL and WASO. The reason for this may be timing of the data capture as the electronic entry was always within 18 hours of waking that morning.

## 5. Study Limitations

This study sample was healthy community dwelling residents; thus data from this study should not be inferred to populations with significant comorbidities. Thirty percent of the participants in this study reported working evenings or night shifts at least on occasion. The shift they were working during study procedures was not collected. Participants were instructed to fill out the paper and electronic diary twice a day. Electronic capture was time-stamped but there was no way to detect when the paper diary was filled out. Thus some participants could have filled both out at the same time using the exact same numbers. This is an observation study, without a control condition, thus weaker design.

## 6. Conclusions

The use of a wrist-worn device to capture daily sleep diary data is feasible and results in more precise and interpretable data as compared to capture via a paper diary. With the advent of electronic devices and proclivity for their use, it is important to be assured that the data captured is as accurate as the traditional paper diary capture. The device used in this study (PRO-diary) has the added advantage of also capturing objective sleep data via actigraphy. Other advantages of electronic capture are the ability to time-stamp the entry, increased legibility, and lack of need for data entry from paper capture. Downfalls of the device used in this study were the inability to aggregate all participants' data automatically and lack of communication between the diary software and the motion detection software within the device.

## Figures and Tables

**Table 1:** Participant characteristics (*n* = 35).

Sex (female)—*n* (%)	26 (74.3)
Age (years)—mean (sd)	36 (15)
Race—*n* (%)	
African American	5 (14.3)
Caucasian	28 (80.8)
Mixed	1 (2.9)
Hispanic or Latino	1 (2.9)
Education—*n* (%)	
High school	4 (11.4)
College 2 years	8 (22.9)
College 4 years	13 (37.1)
College > 4 years	10 (28.6)
Household income ($/year)—*n* (%)	
<30,000	11 (31.4)
30,000–50,000	3 (8.6)
50,000–100,000	16 (45.7)
>100,000	2 (5.7)
Shift worker (day/eve. or day/night)—*n* (%)	11 (31)
PROMIS^a^—norm mean	
Physical function	55 (6)
Anxiety	48 (9)
Depression	45 (9)
Fatigue	48 (9)
Sleep	48 (9)
Social role satisfaction	53 (9)
Pain interference	48 (7)
Sleep measures—mean (sd)	
Insomnia Severity Index	7 (5)
Epworth sleepiness scale	7 (4)
AHI	2.5 (3)

*Notes*. ^a^PROMIS measures standardized to population with mean = 50, sd 10.

AHI—apnea hypopnea index.

**Table 2:** Diary and actigraphy measures of sleep latency, waking after sleep onset, and number of awakenings during sleep.

	Diary assessments mean (sd)		Actigraphy measures mean (sd)
	Paper	Electronic	
SL (minutes)	19 (20)	23 (22)	29 (19)
WASO (minutes)	4 (6)	15 (13)	66 (28)
NWAK	1.5 (2)	1.8 (1.4)	52 (14)
TST (minutes)	412 (97)	434 (50)	398 (58)
SE (%)	90 (.1)	92 (.05)	83 (6)

NWAK—number of awakenings during sleep, SL—sleep latency, WASO—waking after sleep onset, SE—sleep efficiency, and TST—total sleep time.

**Table 3:** Descriptive and correlations of actigraphy and electronic diary data.

	Actigraphy mean (sd)	Electronic diary mean (sd)	Pearson's *r*
SL (minutes)	29 (19)	23 (21)	.44^*∗∗*^
WASO (minutes)	66 (28)	15 (13)	.41^*∗*^
NWAK	52 (14)	1.8 (1.4)	−.08
TST (minutes)	398 (58)	434 (50)	.64^*∗∗*^
SE (%)	83 (6)	92 (.05)	.20

^*∗*^ *p* < .05.

^*∗∗*^ *p* < .01.

NWAK—number of awakenings during sleep, SL—sleep latency, and WASO—waking after sleep onset.

**Table 4:** Correlation of actigraphy and paper diary data (*n* = 35).

	Actigraphy mean (sd)	Paper diary mean (sd)	Pearson's *r*
SL (minutes)	29 (19)	18 (19)	.15
WASO (minutes)	66 (28)	4 (6)	.22
NWAK	52 (14)	1.5 (1)	−.15
TST (minutes)	398 (58)	412 (97)	.39^*∗*^
SE (%)	83 (6)	90 (.1)	.05

^*∗*^ *p* < .05.

NWAK—number of awakenings during sleep, SL—sleep latency, WASO—waking after sleep onset, SE—sleep efficiency, and TST—total sleep time.
